# Correlates of screen-based behaviors among adults from the 2019 Brazilian National Health Survey

**DOI:** 10.1186/s12889-021-12340-0

**Published:** 2021-12-15

**Authors:** Danilo R. Silva, Paul Collings, Raphael H. O. Araujo, Luciana L. Barboza, Célia L. Szwarcwald, André O. Werneck

**Affiliations:** 1 Department of Physical Education, Federal University of Sergipe – UFS, Avenida Marechal Rondon, s/no, Rosa Elze, São Cristóvão, SE CEP 49100-000 Brazil; 2 Bradford Institute for Health Research, Bradford Teaching Hospitals NHS Foundation Trust, Bradford, UK; 3 Graduation Program in Health Sciences, Londrina State University, Londrina, Brazil; 4 Graduation Program in Physical Education, University of Brasília (UnB), Brasília, Brazil; 5 Instituto de Comunicação e Informação Científica e Tecnológica em Saúde (ICICT), Fundação Oswaldo Cruz (Fiocruz), Rio de Janeiro, Brazil; 6 Center for Epidemiological Research in Nutrition and Health, Department of Nutrition, School of Public Health, University of São Paulo (USP), São Paulo, Brazil

**Keywords:** Sedentary behavior, Screen time, Television viewing, Adults, Brazil

## Abstract

We aimed to investigate correlates of TV viewing and other types of screen-based behaviors in a nationally representative sample of Brazilian adults. In the 2019 Brazilian National Health Survey (including 88,509 adults), TV viewing time and other types of screen behaviors (computer, tablet, and cellphone use) were self-reported and different geographical, sociodemographic, behavioral, and health status factors were investigated as potential correlates. Multinomial logistic regression models were used for the main analyses. Living in capital cities, urban areas, being unemployed, high consumption of soft drinks, obesity, and elevated depressive symptoms were each associated with more TV viewing and more time using other types of screens. There were differential associations between TV viewing and the use of other types of screen across age and socioeconomic variables. For instance, younger adults have a more diverse portfolio of screen time than older adults. To conclude, levels of screen-based behaviors vary by geographical, sociodemographic, behavioral, and health status characteristics. Interventions should focus on high-risk population groups and may benefit from targeting specific sedentary behaviors of interest.

**Supplementary Information:**

The online version contains supplementary material available at 10.1186/s12889-021-12340-0.

## Introduction

Sedentary behavior represents a considerable public health challenge [1]. Excessive sedentary time increases the risk of morbidity and mortality [2, 3], especially in individuals who are concurrently physically inactive [4], which represents 27.5% of the population [5]. National and international organizations have published guidelines and public health policies which recommend reductions in sedentary behavior, and that endorse population-level surveillance systems [6–9]. Regardless, a high prevalence of sedentary behaviors persists in different parts of the world, and relative to research conducted within the physical activity realm, surveys on sedentary behaviors at the population level are limited by lack of coverage, being available especially from high-income countries [10, 11].

By definition [12], sedentary behaviors can happen in a range of contexts and domains of life (e.g. reading, writing, using electronic devices), and different behaviors may have a unique profile of correlates and each may be differently associated with health [13]. National health surveys have usually assessed sedentary behavior using crude methods. For example, they have captured limited information about sedentary behaviors as part of questionnaires that have otherwise focused on physical activity (e.g. surveys have captured sitting time by the International Physical Activity Questionnaire [IPAQ] or the Global Physical Activity Questionnaire [GPAQ]), or they have focused only on screen time, especially television (TV) viewing. For these reasons, most of the available evidence about the prevalence, temporal trends, and correlates of sedentary behaviors are about total sitting time and TV viewing [10, 14–16].

These concepts are particularly important considering that there is evidence of an emerging “screen transition”, with individuals gradually spending less time watching TV and more time engaged with other screens [17]. Providing an enhanced understanding about the detailed correlates of TV viewing, and simultaneously the detailed correlates of other types of screens, could help to assist the development of effective interventions that can be used to limit increases, or target reductions, in specific screen-based behaviors.

To date, studies that have investigated sedentary behavior correlates have consistently found that individual characteristics, including sex, age, illness, other lifestyle behaviors (e.g. tobacco smoking, diet), and sociodemographic factors (e.g. education) are associated with the amount of leisure-time sedentary behavior [18–20]. On the other hand, whilst there is some evidence about the correlates of recreational screen-based behaviors, especially TV viewing, few national surveys have evaluated other types of screen-based behaviors, such as the use of computers, tablets, and cellphones. We aimed to investigate the correlates of different types of screen-based behaviors in a national sample of Brazilian adults.

## Methods

### Design and sample

This study used data from the 2019 Brazilian National Health Survey, a cross-sectional study conducted with a nationally representative sample of adults in Brazil. The sampling process occurred through three stages. First, census tracts were randomly selected; second, households were randomly selected; third, in each household one inhabitant (aged ≥15y old) was randomly selected. From 100,541 selected households, interviews were conducted with a total of 94,114 participants. In this complete-case analysis, participants aged < 18 years and those with missing data for variables of interest were excluded. The final study sample was composed of 88,509 adults. Further methodological details about the survey are available elsewhere [21]. The Brazilian Council of Ethics in Research approved all procedures according to the Helsinki declaration.

### Screen-based behaviors

Participants self-reported the daily time they usually spent engaged in screen-based behaviors. TV viewing was assessed using the question: “*How many hours a day do you usually spend watching TV?*”. The other types of screen-based behavior were assessed through the question: “*How many hours in your daily free time do you usually use a computer, tablet, or cellphone, to access social media, news, videos, games, etc?*”. For both questions the possible responses were: a) less than 1 h/d; b) more than 1 h/d but less than 2 h/d; c) more than 2 h/d but less than 3 h/d; d) more than 3 h/d but less than 6 h/d; e) more than 6 h/d; and f) do not use. For analysis purposes screen time was reclassified into four categories: None (0 h/d) / Typical (> 0 to < 3 h/d) / Moderate (≥3 to < 6 h/d) / High (≥6 h/d). This classification was based on the Canadian 24 h Movement Guidelines [8], which suggests < 3 h of recreational screen time per day for adults. In addition, we created a further category of no use, given the specific profiles of non-users of TV-viewing [22], and another category of excessive behavior (twice the recommendation) for sensitivity analyses.

### Correlates

A range of correlates across four dimensions was investigated. Possible geographical correlates included the macro regions of the country (North, Northeast, Southeast, South, and Midwest), type of city (capital or non-capital of the State), and characteristics of the surrounding area (urban or rural). Demographic factors included sex (men/women), age group (18-34y, 35-49y, 50-64y, and ≥ 65y), highest academic attainment (up to high school, high school, and college or more), income (equivalent to ≤1 times the minimum wage, 1–3 times the minimum wage, and > 3 times the minimum wage), and internet access (yes/no). The sociodemographic variables were classified based on previous studies from the Brazilian National Health Survey [23, 24].

We also investigated other lifestyle behaviors as correlates, including self-reported leisure-time physical activity (< 150 min/week versus ≥150 min/week, according to the current guidelines from the World Health Organization [6]), consumption of sugary foods and soft drinks (both classified as < 5 days/week versus ≥5 days/week, based on the frequent consumption [25]). Finally, health status factors, comprising elevated depressive symptoms (defined as a score > 9 in the Patient Health Questionnaire-9 [26]), obesity (defined as a body mass index ≥30 kg/m^2^ [27]), and self-rated health (good/bad [28]) were also investigated as screen time correlates.

### Statistical procedures

We used percentages values and 95% confidence intervals to describe the distribution of each group of correlates according to screen time categories. For the main analyses, multinomial logistic regression models were created. Models included each of the groups of geographical, demographic, other lifestyle behaviors, and health status correlates in turn. Variables were tested at each level and only individual correlates that were statistically significantly associated with screen time (*p*-value < 0.05) were included in a final model. Estimates were weighted considering the characteristics of the general population as well as the non-response rate [21]. All analyses were conducted in Stata 15, considering sample weights (survey command).

## Results

The characteristics of the final sample are presented in Table 1. Of the final sample, 53.2% were women. The weighted prevalence of high (≥6.0 h/d) TV viewing and other types of screen-based behavior were 5.8 and 8.6%, respectively. While 8.6% reported no TV viewing, 27.3% reported no use of other types of screen-based behavior. The unadjusted prevalence of TV viewing and other types of screen-based behavior are presented in Supplementary Tables 1 and 2, respectively.

**Table 1:** Characteristics of the sample (*n* = 88,509)

	%	95%CI
**Geographical factors**		
**Region**		
North	7.9	7.7–8.1
Northeast	26.4	26.1–26.9
Southeast	43.4	42.8–44.1
South	14.7	14.3–15.0
Midwest	7.6	7.4–7.8
**Type of city**		
Capital	41.6	41.1–42.2
Others	58.4	57.8–58.9
**Type of residence**		
Urban	86.2	85.9–86.5
Rural	13.8	13.5–14.1
**Demographic factors**		
**Sex**		
Men	46.8	46.3–47.4
Women	53.2	52.6–53.7
**Age group**		
18-34y	32.3	31.4–32.5
35-49y	29.3	28.8–29.8
50-64y	22.6	22.1–23.1
≥ 65y	16.1	15.7–16.5
**Highest academic achievement**		
Up to high school	49.2	48.7–30.4
High school	29.8	29.2–30.4
College or more	21.0	20.5–21.4
**Employment status**		
Employed	56.2	55.6–56.8
Unemployed	43.8	43.2–44.4
**Income**		
≤ 1 times minimum wage	51.2	50.6–51.8
1–3 times minimum wage	37.3	36.7–37.9
> 3 times minimum wage	11.5	11.2–11.9
**Internet access**		
Yes	84.6	84.2–84.9
No	15.4	15.1–15.8
**Lifestyle behaviors**		
**Leisure physical activity**		
< 150 min/week	73.5	73.0–74.0
≥ 150 min/week	26.5	26.0–27.0
**Sugary foods consumption**		
< 5 days/week	85.2	84.7–85.6
≥ 5 days/week	14.8	14.4–15.3
**Soft drink consumption**		
< 5 days/week	90.8	90.4–91.1
≥ 5 days/week	9.2	8.9–9.6
**Health status**		
**Elevated depressive symptoms**		
No	89.2	88.8–89.5
Yes	10.8	10.5–11.2
**Obesity**		
No	78.0	77.4–78.5
Yes	22.0	21.5–22.6
**Self-rated health**		
Good	66.1	65.6–66.6
Bad	33.9	33.4–34.4
**Outcomes**		
**TV viewing**		
None (0 h/d)	8.6	8.3–9.0
Typical (> 0 to < 3 h/d)	69.6	69.0–70.1
Moderate (≥3.0 to < 6 h/d)	15.9	15.5–16.4
High (≥6.0 h/d)	5.8	5.6–6.1
**Other screen-based behaviors**^a^		
None (0 h/d)	27.3	26.8–27.7
Typical (> 0 to < 3 h/d)	50.6	50.0–51.1
Moderate (≥3.0 to < 6 h/d)	13.6	13.1–14.0
High (≥6.0 h/d)	8.6	8.3–9.0

Elevated depressive symptoms are defined as a score > 9 in the Patient Health Questionnaire-9. Obesity is defined as a body mass index ≥30 kg/m^2^

*CI* confidence interval

^a^Computer, tablet, or cellphone use to access social media, news, videos, games, etc.

Results of the adjusted multinomial logistic regression models, which were used to identify the main correlates of both TV viewing and other screen-based behaviors, are shown in Tables 2 and 3, respectively. Table 2 shows that higher odds of moderate and high TV viewing were associated with living in the Southeast macro region (compared to the North), being aged ≥65y, unemployment, high soft drink consumption, and obesity. In addition, higher odds of high TV viewing was associated with living in the Northeast macro region (compared to North), and having depressive symptoms. Conversely, lower odds of moderate TV viewing was associated with living in the Midwest macro region (compared to North) and having no internet access, and lower odds of both moderate and high TV viewing was associated with living in non-capitals cities and rural areas, being aged 35-49y, and having higher educational attainment. Factors associated with higher likelihood of not watching TV included living in non-capital cities, being a woman, having the highest level of academic attainment, no internet access, and depressive symptoms. Older age and higher incomes were associated with lower odds of watching no TV.

**Table 2:** Adjusted regression model quantifying the correlates of TV viewing in Brazilian adults

	TV viewing			
	None (0 h/d) % (95%CI)	Typical (> 0 to < 3 h/d) % (95%CI)	Moderate (≥3.0 to < 6 h/d) % (95%CI)	High (≥6.0 h/d) % (95%CI)
**Geographical factors**				
**Region**				
North	1	Ref	1	1
Northeast	0.90 (0.82–1.00)	Ref	1.05 (0.96–1.14)	**1.32 (1.15–1.50)**
Southeast	0.86 (0.76–0.98)	Ref	**1.17 (1.06–1.29)**	**1.50 (1.31–1.73)**
South	0.74 (0.65–0.85)	Ref	0.99 (0.89–1.11)	0.97 (0.81–1.16)
Midwest	1.05 (0.92–1.20)	Ref	**0.87 (0.77–0.97)**	0.99 (0.82–1.18)
**Type of city**				
Capital	1	Ref	1	1
Others	**1.22 (1.11–1.34)**	Ref	**0.82 (0.76–0.88)**	**0.55 (0.49–0.62)**
**Type of residence**				
Urban	1	Ref	1	1
Rural	0.92 (0.83–1.01)	Ref	**0.75 (0.69–0.82)**	**0.56 (0.48–0.65)**
**Demographic factors**				
**Sex**				
Men	1	Ref	1	1
Women	**1.11 (1.01–1.23)**	Ref	1.00 (0.93–1.07)	1.00 (0.89–1.12)
**Age group**				
18-34y	1	Ref	1	1
35-49y	**0.62 (0.55–0.69)**	Ref	**0.89 (0.80–0.97)**	**0.76 (0.65–0.89)**
50-64y	**0.54 (0.48–0.61)**	Ref	0.99 (0.91–1.10)	0.90 (0.77–1.05)
≥ 65y	**0.68 (0.60–0.78)**	Ref	**1.34 (1.20–1.50)**	**1.21 (1.03–1.43)**
**Highest academic achievement**				
Up to high school	1		1	1
High school	0.98 (0.87–1.10)	Ref	1.09 (1.00–1.19)	**0.81 (0.71–0.92)**
College or more	**1.24 (1.08–1.43**)	Ref	**0.69 (0.62–0.78)**	**0.44 (0.36–0.53)**
**Employment status**				
Employed	1	Ref	1	1
Unemployed	1.07 (0.97–1.18)	Ref	**1.58 (1.46–1.70)**	**3.02 (2.64–3.45)**
**Income**				
≤ 1 times minimum wage	1	Ref	1	1
1–3 times minimum wage	**0.80 (0.71–0.89)**	Ref	1.02 (0.95–1.12)	1.00 (0.87–1.14)
> 3 times minimum wage	**0.68 (0.57–0.82)**	Ref	0.97 (0.85–1.10)	0.98 (0.81–1.19)
**Internet access**				
Yes	1	Ref	1	1
No	**1.35 (1.22–1.48)**	Ref	**0.91 (0.84–0.99)**	1.04 (0.92–1.18)
**Lifestyle behaviors**				
**Soft drink consumption**				
< 5 days/week	1	Ref	1	1
≥ 5 days/week	1.03 (0.87–1.22)	Ref	**1.31 (1.16–1.48)**	**1.55 (1.31–1.85)**
**Health status**				
**Elevated depressive symptoms**				
No	1	Ref	1	1
Yes	**1.81 (1.59–2.07)**	Ref	1.00 (0.90–1.11)	**1.67 (1.45–1.93)**
**Obesity**				
No	1	Ref	1	1
Yes	0.96 (0.85–1.09)	Ref	**1.24 (1.14–1.32)**	**1.46 (1.31–1.64)**

The final model is adjusted for all variables presented. Variable with *p* > 0.05 was removed from the final model. The data are odds ratios and indicate that, for example, compared to participants in urban areas those in rural areas were 44% less likely to be in the high TV viewing than the typical TV viewing group

**Table 3:** Adjusted regression model quantifying the correlates of screen time (except TV viewing) in Brazilian adults

	Computer, tablet, or cellphone use to access social media, news, videos, games, etc.			
	None (0 h/d) % (95%CI)	Typical (> 0 to < 3 h/d) % (95%CI)	Moderate (≥3.0 to < 6 h/d) % (95%CI)	High (≥6.0 h/d) % (95%CI)
**Geographical factors**				
**Region**				
North	1	Ref	1	1
Northeast	**0.88 (0.81–0.96)**	Ref	0.99 (0.89–1.10)	1.14 (1.00–1.30)
Southeast	**0.56 (0.51–0.62)**	Ref	0.92 (0.82–1.04)	0.91 (0.79–1.05)
South	**0.58 (0.52–0.65)**	Ref	**0.79 (0.69–0.89)**	**0.78 (0.66–0.92)**
Midwest	**0.55 (0.49–0.63)**	Ref	0.94 (0.83–1.07)	0.90 (0.76–1.06)
**Type of city**				
Capital	1	Ref	1	1
Others	**1.25 (1.16–1.35)**	Ref	**0.87 (0.80–0.95)**	**0.69 (0.62–0.76)**
**Type of residence**				
Urban	1	Ref	1	1
Rural	**1.94 (1.79–2.09)**	Ref	**0.76 (0.67–0.86)**	**0.54 (0.45–0.63)**
**Demographic factors**				
**Sex**				
Men	1	Ref	1	1
Women	**0.71 (0.66–0.77)**	Ref	1.01 (0.93–1.10)	0.93 (0.84–1.03)
**Age group**				
18-34y	1	Ref	1	1
35-49y	**2.03 (1.84–2.24)**	Ref	**0.41 (0.37–0.45)**	**0.27 (0.24–0.30)**
50-64y	**4.54 (4.10–5.03)**	Ref	**0.25 (0.23–0.29)**	**0.14 (0.12–0.16)**
≥ 65y	**12.61 (11.20–14.19)**	Ref	**0.21 (0.18–0.26)**	**0.11 (0.08–0.14)**
**Highest academic achievement**				
Up to high school	1	Ref	1	1
High school	**0.44 (0.40–0.47)**	Ref	**1.37 (1.24–1.51)**	**1.20 (1.06–1.35)**
College or more	**0.22 (0.19–0.25)**	Ref	**1.26 (1.12–1.43)**	1.01 (0.87–1.16)
**Employment status**				
Employed	1	Ref	1	1
Unemployed	**1.55 (1.43–1.67)**	Ref	**1.18 (1.08–1.32)**	**1.74 (1.56–1.95)**
**Income**				
≤ 1 times minimum wage	1	Ref	1	1
1–3 times minimum wage	**0.64 (0.59–0.69)**	Ref	**1.19 (1.07–1.31)**	**1.14 (1.01–1.28)**
> 3 times minimum wage	**0.48 (0.42–0.56)**	Ref	1.10 (0.96–1.28)	1.09 (0.90–1.31)
**Internet access**				
Yes	1	Ref	1	1
No	**5.90 (5.45–6.40)**	Ref	**0.60 (0.50–0.71)**	**0.54 (0.43–0.66)**
**Lifestyle behaviors**				
**Leisure physical activity**				
< 150 min/week	1	Ref	1	1
≥ 150 min/week	**1.54 (1.42–1.67)**	Ref	**0.88 (0.80–0.96)**	**0.84 (0.76–0.94)**
**Sugary foods consumption**				
< 5 days/week	1	Ref	1	1
≥ 5 days/week	0.98 (0.89–1.09)	Ref	**1.19 (1.06–1.35)**	**1.44 (1.28–1.63)**
**Soft drink consumption**				
< 5 days/week	1	Ref	1	1
≥ 5 days/week	1.12 (0.97–1.29)	Ref	**1.40 (1.22–1.60)**	**2.06 (1.79–2.37)**
**Health status**				
**Elevated depressive symptoms**				
No	1	Ref	1	1
Yes	**1.13 (1.01–1.26)**	Ref	**1.34 (1.10–1.63)**	**1.55 (1.34–1.80)**
**Obesity**				
No	1	Ref	1	1
Yes	**0.90 (0.83–0.97)**	Ref	**1.19 (1.08–1.32)**	**1.16 (1.04–1.30)**
**Self-rated health**				
Good	1	Ref	1	1
Bad	**1.25 (1.16–1.34)**	Ref	**0.88 (0.80–0.97)**	1.09 (0.97–1.24)

The final model is adjusted for all variables presented. Variable with p > 0.05 was removed from the final model. The data are odds ratios and indicate that, for example, compared to participants without elevated depressive symptoms those with elevated depressive symptoms were 55% less likely to be in the high screen time than the typical screen time group

Table 3 shows that higher likelihood of moderate and high use of other screen types was associated with intermediate educational attainment (compared to the lowest educational status), unemployment, incomes 1–3 times higher than the minimum wage, high consumption of sugary foods, high intake of soft drinks, depressive symptoms, and obesity. In addition, a higher likelihood of moderate use of other screens was associated with the highest level of academic attainment, whereas participants with good self-rated health displayed lower odds of moderate use. In contrast, lower odds of moderate and high use of other types of screen-based behaviors was associated with living in the South macro region (compared to the North), non-capital cities, and rural areas, older age, having no internet access, and physical activity. Except for regional differences, the same factors were associated with higher likelihood of not using other screen types, and the association was strongest for adults aged ≥65y. Other factors associated with higher likelihood of not using other screen types were unemployment, depressive symptoms, and bad self-rated health. Factors associated with lower odds of not using other screen types included living in all macro regions compared to North, being a woman, having higher academic attainment, higher income, and obesity.

## Discussion

This investigation shows that living in capital cities, urban areas, being unemployed, poor dietary behaviors, obesity, and elevated depressive symptoms were consistently associated with higher screen time, regardless of type. There were differential associations between TV viewing and use of other types of screen-based behavior across age and socioeconomic variables. Younger adults have a more diverse portfolio of screen time than older adults.

Regarding TV viewing, our results concur with existing evidence, which has shown that higher TV viewing is correlated with lower educational status, poorer dietary habits, and negative health outcomes [29–31]. The geographical distribution of TV viewing has seldom been studied. We observed that Brazilian adults living in the Northeast and Southeast macro regions were more likely to spend excess time watching TV. In addition, we found that adults living in non-capital cities and rural areas were less likely to watch higher volumes of TV and were also less likely to engage in higher usage of other types of screen-based behavior; in general, they were more likely not to use any screens at all. These results may be related to limited internet availability in rural locations, indeed we found that having no internet access was markedly associated with higher odds of not using other types of screen-based behavior. This pattern of results may also be explained by greater availability and accessibility of public parks and green spaces, lower violence and fewer issues of perceived safety in smaller non-capital cities and rural landscapes [32].

We also identified that unemployment was associated with higher likelihood of moderate and high TV viewing. A u-shaped association was apparent for other types of screen-based behavior, such that unemployment was associated both with higher likelihood of moderate and high use of other types of screen-based behavior, and also with higher odds of not using any other types of screen-based behavior at all. This may be explained by unemployed younger adults using diverse types of screen device throughout the day, and older retirees not using other types of screen. Taken together, the results highlight priority groups that may benefit most from interventions that are designed to reduce screen time.

The main novelty of this study was that we were able to contrast the correlates of TV viewing (which is an important sedentary behavior in its own right and an often used, albeit inadequate, proxy for total sedentary time) with that of other screen types that are increasingly prevalent worldwide [33]. We observed different associations for TV viewing and other screen types across age and socioeconomic variables. For instance, higher educational attainment was associated with lower odds of moderate and high TV viewing, but with higher odds of moderate and high use of all other screens. In addition, the oldest group of adults was more likely to watch moderate and high volumes of TV, but, compared to the youngest age group, older adults of all other ages were less likely to engage in moderate or high usage of other screens, and were more likely to report not using any other screens at all. This pattern of results may reflect social, cultural, and economic differences in the accessibility and usability of different types of screen-based device. The results corroborate previous studies and provide additional support for the concept of an emerging “screen transition”, which has previously been shown to be influenced by socioeconomic factors and technological shifts in low- and middle-income countries [17, 31, 34, 35].

It is conceivable that sedentary behaviors may be associated with higher adiposity and adverse health not because of sedentary time per se, but because of coexisting (possibly mediating) obesogenic diet, inactivity, and sleep behaviors [24, 36]. Accordingly, we found that a higher intake of soft drinks was associated with higher TV viewing and higher use of other types of screen-based behavior. Higher consumption of sugary foods was further associated with higher use of other types of screens. The coexistence of TV viewing with poor dietary habits is well established and our findings add to emerging evidence-base for other types of screen-based behavior [37, 38]. We also found being more physically active was associated with lower odds of moderate and high use of other screen types, and with higher odds of not using any other screen types at all.

Regarding health status, obesity and elevated depressive symptoms were associated with higher volumes of TV viewing and also with higher use of other types of screen-based behavior. There was a difference in the shape of associations, however, with some evidence that the associations for depressive symptoms were curvilinear (depressive symptoms were also associated with higher likelihood of not watching TV and not using other screen types). Unfortunately, because this is a cross-sectional study, it is impossible to assign any direction of association to our results. This is particularly problematic for obesity and depressive symptoms, which may exhibit bidirectional associations with sedentary behaviors [39, 40]. Obesity and depressive symptoms may be both a cause and a consequence of higher TV time and higher use of other screens.

As far as we are aware, this is the first study to explore correlates of different types of screen-based behaviors in a representative sample of Brazilian adults. It is advantageous that we investigated TV viewing and other screens, as well as myriad potential correlates that spanned diverse dimensions. By doing so we have provided an enhanced understanding about the distributions of screen use throughout the Brazilian adult population. This information can be used to assist the development of targeted and more effective interventions to reduce screen-based behaviors in high-risk population groups, such as health education, counseling, household environmental changes and using strategies of usage control by the own devices [41, 42]. A limitation of the current study includes the joint analysis of multiple types of screen-based behavior (computer, tablet, and cellphones) which precluded a more refined analysis of specific screen-based behaviors. It is also a weakness that all data were self-reported, meaning they are subject to inaccurate and biased responses.

To conclude, TV viewing and other screen types share certain correlates among Brazilian adults, however, there are unique screen-type specific correlates. The main differences among the screen types were observed for socioeconomic status and age groups.

## Supplementary Information

**Additional file 1.**

## Data Availability

Data is available under open access through the link: https://www.ibge.gov.br/estatisticas/sociais/saude/9160-pesquisa-nacional-de-saude?=&t=microdados. Accession number: PNS 2019 (*Arquivos de Microdados da PNS 2019*).

## Abbreviations

TV: Television
IPAQ: International Physical Activity Questionnaire
GPAQ: Global Physical Activity Questionnaire
