# Supplementary material for: Correlates of screen-based behaviors among adults from the 2019 Brazilian National Health Survey
Source: BMC Public Health. 2021 Dec 15;21:2289. doi: 10.1186/s12889-021-12340-0 (PMC8672534; doi:10.1186/s12889-021-12340-0)
Supplement: Supplementary file 1 — Additional file 1. [file 12889_2021_12340_MOESM1_ESM.docx]

**Supplementary table 1.** Unadjusted prevalence of TV viewing categories according to potential correlates in Brazilian adults.

|  | **TV viewing** | | | |
| --- | --- | --- | --- | --- |
|  | 0 h/d  % (95%CI) | >0 to <3 h/d  % (95%CI) | ≥3.0 to <6 h/d  % (95%CI) | ≥6.0 h/d  % (95%CI) |
| **Geographical factors** |  |  |  |  |
| **Region** |  |  |  |  |
| North | 10.7 (10.0-11.5) | 70.1 (69.0-71.2) | 14.6 (13.8-15.5) | 4.5 (4.1-5.0) |
| Northeast | 9.6 (9.1-10.1) | 69.4 (68.6-70.2) | 15.1 (14.5-15.8) | 5.9 (5.5-6.5) |
| Southeast | 7.9 (7.3-8.5) | 68.0 (66.9-69.0) | 17.4 (16.5-18.2) | 6.8 (6.3-7.3) |
| South | 7.4 (6.7-8.1) | 73.4 (72.2-74.5) | 15.1 (14.2-16.0) | 4.2 (3.7-4.8) |
| Midwest | 10.2 (9.3-11.2) | 71.5 (70.2-72.8) | 13.4 (12.5-14.4) | 4.8 (4.2-5.5) |
| **Type of city** |  |  |  |  |
| Capital | 7.4 (7.0-7.8) | 67.4 (66.7-68.2) | 17.4 (16.9-18.0) | 7.7 (7.3-8.1) |
| Others | 9.5 (9.1-10.1) | 71.1 (70.3-71.9) | 14.8 (14.2-15.5) | 4.5 (4.2-4.9) |
| **Type of residence** |  |  |  |  |
| Urban | 8.4 (8.1-8.8) | 68.9 (68.3-69.5) | 16.5 (16.0-17.0) | 6.2 (5.9-6.5) |
| Rural | 10.1 (9.5-10.7) | 73.9 (73.0-74.9) | 12.5 (11.9-13.3) | 3.4 (3.0-3.9) |
| **Demographic factors** |  |  |  |  |
| **Sex** |  |  |  |  |
| Men | 8.0 (7.6-8.6) | 71.4 (70.6-72.2) | 15.5 (14.8-16.1) | 5.1 (4.7-5.5) |
| Women | 9.2 (8.7-9.7) | 67.9 (67.2-68.7) | 16.3 (15.8-16.9) | 6.6 (6.2-6.9) |
| **Age group** |  |  |  |  |
| 18-34y | 11.7 (10.9-12.5) | 68.1 (67.0-69.2) | 14.9 (14.1-15.8) | 5.3 (4.8-5.9) |
| 35-49y | 7.7 (7.2-8.3) | 74.4 (73.4-75.3) | 13.8 (13.1-14.6) | 4.1 (3.7-4.5) |
| 50-64y | 6.4 (5.9-7.0) | 71.4 (70.4-72.4) | 16.2 (15.4-17.0) | 6.0 (5.5-6.5) |
| ≥65y | 7.5 (6.9-8.2) | 61.2 (60.0-62.4) | 21.4 (20.3-22.4) | 9.9 (9.2-10.7) |
| **Highest academic achievement** |  |  |  |  |
| Up to high school | 7.9 (7.5-8.4) | 67.9 (67.1-68.8) | 16.8 (16.2-17.5) | 7.3 (6.8-7.8) |
| High school | 8.1 (7.5-8.7) | 68.4 (67.4-69.4) | 17.8 (17.0-18.9) | 5.7 (5.3-6.2) |
| College or more | 8.3 (7.4-9.2) | 74.4 (73.4-76.0) | 13.5 (12.5-14.5) | 3.5 (3.0-4.1) |
| **Employment status** |  |  |  |  |
| Employed | 8.6 (8.1-9.1) | 74.9 (74.2-75.6) | 13.4 (12.8-14.0) | 3.1 (2.8-3.4) |
| Unemployed | 8.7 (8.3-9.2) | 62.8 (61.9-63.6) | 19.2 (18.5-19.8) | 9.4 (8.9-9.9) |
| **Income** |  |  |  |  |
| ≤1 times minimum wage | 10.0 (9.2-10.8) | 71.7 (70.4-72.9) | 13.6 (12.7-14.5) | 4.8 (4.2-5.4) |
| 1-3 times minimum wage | 8.5 (7.8-9.2) | 74.2 (73.1-75.2) | 14.2 (13.4-15.0) | 3.2 (2.8-3.6) |
| > 3 times minimum wage | 6.5 (5.8-7.3) | 80.0 (78.5-81.4) | 11.7 (10.5-13.0) | 2.8 (1.4-2.2) |
| **Internet access** |  |  |  |  |
| Yes | 8.3 (7.9-8.7) | 70.1 (69.5-70.7) | 16.0 (15.5-16.5) | 5.6 (5.3-5.9) |
| No | 10.6 (10.0-11.3) | 66.7 (65.6-67.7) | 15.6 (14.8-16.4) | 7.1 (6.6-7.7) |
| **Lifestyle behaviors** |  |  |  |  |
| **Leisure physical activity** |  |  |  |  |
| <150 min/week | 8.5 (8.2-9.0) | 68.8 (68.2-69.4) | 16.1 (15.7-16.6) | 6.4 (6.1-6.8) |
| ≥150 min/week | 8.8 (8.2-9.5) | 71.6 (70.5-72.8) | 15.3 (14.4-16.3) | 4.2 (3.7-4.7) |
| **Sugary foods consumption** |  |  |  |  |
| <5 days/week | 8.7 (8.4-9.1) | 70.0 (69.4-70.6) | 15.6 (15.2-16.1) | 5.6 (5.3-5.9) |
| ≥5 days/week | 8.2 (7.4-9.1) | 67.1 (65.5-68.6) | 17.6 (16.3-18.9) | 7.2 (6.4-8.0) |
| **Soft drink consumption** |  |  |  |  |
| <5 days/week | 8.6 (8.3-9.0) | 70.0 (69.4-70.6) | 15.7 (15.2-16.1) | 5.7 (5.4-6.0) |
| ≥5 days/week | 8.7 (7.6-10.0) | 65.3 (63.3-67.2) | 18.4 (16.9-20.1) | 7.5 (6.6-8.6) |
| **Health status** |  |  |  |  |
| **Elevated depressive symptoms** |  |  |  |  |
| No | 8.1 (7.8-8.5) | 70.5 (70.0-71.1) | 16.0 (15.5-16.5) | 5.4 (5.1-5.6) |
| Yes | 13.2 (11.9-14.6) | 61.6 (59.8-63.4) | 15.4 (14.2-16.7) | 9.8 (8.8-10.9) |
| **Obesity** |  |  |  |  |
| No | 8.9 (8.5-9.2) | 70.4 (69.8-71.0) | 15.3 (14.9-15.8) | 5.4 (5.1-5.7) |
| Yes | 7.9 (7.1-8.7) | 66.6 (65.6-67.8) | 18.0 (17.0-19.0) | 7.5 (7.0-8.2) |
| **Self-rated health** |  |  |  |  |
| Good | 8.3 (7.9-8.8) | 71.3 (70.6-72.0) | 15.5 (15.0-16.1) | 4.8 (4.5-5.1) |
| Bad | 9.3 (8.7-9.8) | 66.1 (65.2-67.0) | 16.7 (16.0-17.4) | 7.9 (7.4-8.4) |

**Note.** Elevated depressive symptoms are defined as a score >9 in the Patient Health Questionnaire-9. Obesity is defined as a body mass index ≥30 kg/m^2^. CI, confidence interval.

**Supplementary table 2.** Unadjusted prevalence of screen time categories (except TV viewing) according to potential correlates in Brazilian adults.

|  | **Computer, tablet, or cellphone use to access social media, news, videos, games, etc.** | | | |
| --- | --- | --- | --- | --- |
|  | 0 h/d  % (95%CI) | >0 to <3 h/d  % (95%CI) | ≥3.0 to <6 h/d  % (95%CI) | ≥6.0 h/d  % (95%CI) |
| **Geographical factors** |  |  |  |  |
| **Region** |  |  |  |  |
| North | 35.2 (34.1-36.4) | 43.0 (41.8-44.2) | 13.1 (12.2-14.0) | 8.6 (7.9-9.5) |
| Northeast | 37.4 (36.6-38.2) | 42.2 (41.4-43.1) | 11.8 (11.2-12.4) | 8.6 (8.0-9.1) |
| Southeast | 21.5 (20.7-22.4) | 54.7 (53.6-55.8) | 14.8 (13.9-15.7) | 9.0 (8.4-9.6) |
| South | 25.0 (24.0-26.0) | 55.5 (54.2-56.7) | 12.5 (11.7-13.4) | 7.0 (6.3-7.8) |
| Midwest | 20.8 (19.7-21.9) | 54.1 (52.6-55.5) | 15.5 (14.4-16.6) | 9.6 (8.7-10.7) |
| **Type of city** |  |  |  |  |
| Capital | 19.3 (18.8-19.8) | 53.7 (52.9-54.4) | 15.8 (15.2-16.4) | 11.2 (10.7-11.8) |
| Others | 32.9 (32.2-33.7) | 48.3 (47.5-49.2) | 12.0 (11.4-12.7) | 6.7 (6.3-7.2) |
| **Type of residence** |  |  |  |  |
| Urban | 22.7 (22.2-23.3) | 53.1 (52.5-53.8) | 14.7 (14.2-15.2) | 9.4 (9.1-9.9) |
| Rural | 55.4 (54.3-56.5) | 34.4 (33.4-35.5) | 6.9 (6.2-7.6) | 3.3 (2.9-3.8) |
| **Demographic factors** |  |  |  |  |
| **Sex** |  |  |  |  |
| Men | 28.6 (27.9-29.3) | 49.5 (48.7-50.4) | 13.3 (12.7-14.0) | 8.6 (8.1-9.1) |
| Women | 26.1 (25.5-26.7) | 51.4 (50.7-52.2) | 13.8 (13.2-14.4) | 8.6 (8.2-9.1) |
| **Age group** |  |  |  |  |
| 18-34y | 9.1 (8.6-9.6) | 47.9 (46.7-49.0) | 24.4 (23.4-25.5) | 18.6 (17.7-19.5) |
| 35-49y | 18.8 (18.1-19.6) | 62.5 (61.5-63.5) | 12.6 (11.9-13.4) | 6.0 (5.6-6.5) |
| 50-64y | 35.8 (34.7-36.9) | 54.3 (53.1-55.5) | 6.9 (6.4-7.5) | 2.9 (2.6-3.3) |
| ≥65y | 66.5 (65.3-67.7) | 28.9 (27.7-30.0) | 3.2 (2.7-3.7) | 1.4 (1.2-1.8) |
| **Highest academic achievement** |  |  |  |  |
| Up to high school | 48.1 (47.2-49.1) | 41.7 (40.7-2.6) | 6.4 (5.9-6.8) | 3.8 (3.5-4.2) |
| High school | 13.5 (13.0-14.2) | 56.3 (55.2-57.3) | 18.0 (17.1-18.9) | 12.2 (11.5-12.9) |
| College or more | 5.7 (5.1-6.4) | 67.1 (65.7-68.5) | 17.9 (16.8-19.2) | 9.2 (8.5-10.1) |
| **Employment status** |  |  |  |  |
| Employed | 16.9 (16.3-17.4) | 58.4 (57.8-59.4) | 15.9 (15.2-16.5) | 8.6 (8.2-9.1) |
| Unemployed | 40.6 (39.8-41.4) | 40.2 (39.4-41.0) | 10.7 (10.1-11.3) | 8.6 (8.0-9.1) |
| **Income** |  |  |  |  |
| ≤1 times minimum wage | 30.0 (28.8-31.1) | 47.9 (46.5-49.3) | 13.3 (12.4-14.4) | 8.8 (8.0-9.7) |
| 1-3 times minimum wage | 13.7 (13.0-14.4) | 59.7 (58.6-60.9) | 17.3 (16.3-18.2) | 9.3 (8.7-10.0) |
| > 3 times minimum wage | 6.1 (5.4-7.0) | 70.9 (69.3-72.4) | 15.6 (14.3-16.9) | 7.4 (6.7-8.3) |
| **Internet access** |  |  |  |  |
| Yes | 18.2 (17.8-18.7) | 56.3 (55.7-57.0) | 15.6 (15.1-16.1) | 9.9 (9.5-10.3) |
| No | 76.8 (75.8-77.8) | 18.7 (17.8-19.7) | 2.7 (2.4-3.2) | 1.7 (1.4-2.1) |
| **Lifestyle behaviors** |  |  |  |  |
| **Leisure physical activity** |  |  |  |  |
| <150 min/week | 31.9 (31.4-32.5) | 48.3 (47.6-49.0) | 12.1 (11.6-12.6) | 7.7 (7.3-8.1) |
| ≥150 min/week | 14.2 (13.5-15.0) | 56.8 (55.6-58.0) | 17.8 (16.9-18.8) | 11.1 (10.3-11.9) |
| **Sugary foods consumption** |  |  |  |  |
| <5 days/week | 28.4 (27.9-28.9) | 50.9 (50.3-51.5) | 13.0 (12.5-13.4) | 7.7 (7.4-8.1) |
| ≥5 days/week | 20.6 (19.5-21.9) | 48.7 (47.0-50.3) | 17.1 (15.7-18.6) | 13.5 (12.4-14.7) |
| **Soft drink consumption** |  |  |  |  |
| <5 days/week | 28.4 (27.9-28.9) | 51.1 (50.4-51.7) | 13.0 (12.5-13.4) | 7.6 (7.3-7.9) |
| ≥5 days/week | 16.3 (14.9-17.7) | 45.6 (43.5-47.8) | 19.6 (17.9-21.5) | 18.5 (16.9-20.2) |
| **Health status** |  |  |  |  |
| **Elevated depressive symptoms** |  |  |  |  |
| No | 26.9 (26.4-27.4) | 51.3 (50.7-51.9) | 13.5 (13.1-14.0) | 8.2 (7.9-8.6) |
| Yes | 29.8 (28.3-31.4) | 44.3 (42.9-46.2) | 14.0 (12.5-15.7) | 11.8 (10.7-13.0) |
| **Obesity** |  |  |  |  |
| No | 27.8 (27.3-28.4) | 50.2 (49.5-50.8) | 13.4 (12.9-13.9) | 8.6 (8.2-9.0) |
| Yes | 25.2 (24.2-26.3) | 51.8 (50.5-53.1) | 14.3 (13.3-15.4) | 8.6 (7.9-9.4) |
| **Self-rated health** |  |  |  |  |
| Good | 19.2 (18.7-19.8) | 55.0 (54.2-55.7) | 16.1 (15.5-16.8) | 9.6 (9.2-10.1) |
| Bad | 42.9 (42.0-43.8) | 41.9 (40.9-42.9) | 8.6 (8.0-9.2) | 6.6 (6.1-7.1) |

**Note.** Elevated depressive symptoms defined as a score >9 in the Patient Health Questionnaire-9. Obesity defined as a body mass index ≥30 kg/m^2^. CI, confidence interval.
